# An asymmetric flow-focusing droplet generator promotes rapid mixing of reagents

**DOI:** 10.1038/s41598-021-88174-y

**Published:** 2021-04-22

**Authors:** K. I. Belousov, N. A. Filatov, I. V. Kukhtevich, V. Kantsler, A. A. Evstrapov, A. S. Bukatin

**Affiliations:** 1 Alferov Saint Petersburg National Research Academic University of the Russian Academy of Sciences, Saint Petersburg, Russia; 2 Institute of Silicate Chemistry of RAS, Saint Petersburg, Russia; 3 Institute of Functional Epigenetics, Helmholtz Zentrum München, Neuherberg, Germany; 4 Department of Physics, University of Warwick, Coventry, UK; 5 Institute for Analytical Instrumentation of RAS, Saint Petersburg, Russia

**Keywords:** Lab-on-a-chip, Biomedical engineering, Fluid dynamics, Mechanical engineering

## Abstract

Nowadays droplet microfluidics is widely used to perform high throughput assays and for the synthesis of micro- and nanoparticles. These applications usually require packaging several reagents into droplets and their mixing to start a biochemical reaction. For rapid mixing microfluidic devices usually require additional functional elements that make their designs more complex. Here we perform a series of 2D numerical simulations, followed by experimental studies, and introduce a novel asymmetric flow-focusing droplet generator, which enhances mixing during droplet formation due to a 2D or 3D asymmetric vortex, located in the droplet formation area of the microfluidic device. Our results suggest that 2D numerical simulations can be used for qualitative analysis of two-phase flows and droplet generation process in quasi-two-dimensional devices, while the relative simplicity of such simulations allows them to be easily applied to fairly complicated microfluidic geometries. Mixing inside droplets formed in the asymmetric generator occurs up to six times faster than in a conventional symmetric one. The best mixing efficiency is achieved in a specific range of droplet volumes, which can be changed by scaling the geometry of the device. Thus, the droplet generator suggested here can significantly simplify designs of microfluidic devices because it enables both the droplet formation and fast mixing of the reagents within droplets. Moreover, it can be used to precisely estimate reaction kinetics.

## Introduction

In the past decade droplet microfluidics has been successfully applied to molecular diagnostics for performing highly sensitive assays such as droplet digital polymerase chain reaction (ddPCR)^1^, loop-mediated isothermal amplification (LAMP)^2^, single-cell genome analysis^3–5^ and for detecting and screening enzymes activity^6^. This technique also found a great application in chemical syntheses^7^, especially for syntheses of micro- and nanoparticles with specific requirements^8,9^. The main advantage of droplet microfluidics is in controlled encapsulation of femto- to nanoliters volumes of reaction components. Rapid mixing and negligible thermal inertia within single droplets provide excellent control over reaction conditions, which is ideal for single-cell or single-molecule assays. In addition, the presence of an interface between two immiscible liquids enables a new interfacial synthetic approach^10^.

All these applications usually involve encapsulation of biomolecules, single cells, or polymers with required chemical reagents into individual monodisperse water-in-oil or water-in-oil-in-water droplets. After the encapsulation step biochemical or chemical reactions are performed and followed by, for example, fluorescent detection of the reaction products^11,12^. Due to low deviations in droplet volumes, all the reactions are carried out under uniform conditions, which ultimately lead to the high reproducibility of the assays. In single water-in-oil-emulsions reaction products in most cases are analyzed with custom fluorescent readers or sorters^6,13^. Although, for double emulsions commercial fluorescent activated cell sorters (FACS) can be used^14,15^. In the case of ddPCR, an alternative way to analyze the product is to specifically couple it with magnetic particles, break the emulsion, and make the analysis with FACS^16^.

One of the key points of performing biochemical reactions and analyze their kinetics in microfluidic devices is the effective mixing of reagents^17,18^. Due to laminar flows corresponding to low Reynolds numbers mixing is carried out mainly by diffusion^19,20^. Diffusion associated mixing time scales with the square of the distance, and it can be a limiting factor with increasing the droplet size^21^. This especially might be crucial for high throughput and precise enzyme kinetic measurements for drug screening, point-of-care testing, and investigation of viral fusion^22–24^. Existing approaches are suggested to accelerate mixing in microfluidic devices introducing different types of passive or active mixers. The first type includes microfluidic channels with a special geometry^25,26^ or specific microstructures in them^27^, which facilitate a more efficient interaction of fluids. Active mixers are equipped with various control elements, which require additional energy, such as electric^28^ or mechanical^29^, for operation. While it is challenging to reach high mixing efficiency in passive mixers, active mixers allow to mix reaction components more efficiently but consume energy, and overall device design is more complicated.

In a straight microfluidic channel steady recirculating flows inside moving droplets can enhance mixing, but this is very sensitive to the initial distribution of reagents. This can be achieved by adjusting the relative flow rates of the continuous and dispersed phases in a T-generator^30^. The most common way of mixing, which is insensitive to initial reagents distribution, is to use geometrically induced advection inside droplets. This advection occurs during its motion in a curved microchannel, located just after the droplet formation region^31,32^. Unfortunately, such a curved channel requires additional space and has a cross-section close to droplet size that limits the generation frequency due to its high hydrodynamic resistance.

In this work we propose a novel asymmetric design of flow-focusing droplet generator, which enhances mixing during droplet formation, doesn’t require additional functional elements, and is more compact in comparison with currently available solutions. To define mixing conditions we performed 2D numerical simulations of fluid flows and reagents distribution in droplets and compared obtained results with a conventional symmetric device. The simulations showed that different symmetry of the droplet generator design changes the fluid flows and distribution of reagents in newly formed droplets resulting in different mixing efficiency. Our experimental studies confirmed the simulation results and showed that complete mixing in droplets, formed in the asymmetric droplet generator, occurred significantly faster than in conventional symmetric ones.

## Results and discussion

### Mixing principal

The generation and motion of droplets in a microfluidic device can be described by two-phase flows of two immiscible liquids. Mineral, silicon, or perfluorinated oils are usually used as a continuous phase and water-like liquids as a dispersed phase to produce “water-in-oil” emulsion. In contrast with the no-slip boundary condition on the liquid–solid interface, the liquid–liquid interfaces of such flows are described by velocity and stress continuity conditions^21^:

1 $$\begin{array}{*{20}l} {{\mathbf{Liquid}} - {\mathbf{liquid}}\;{\mathbf{interface}}} \hfill &~~& {{\mathbf{Liquid}}{-}{\mathbf{solid}}\;{\mathbf{interface}}} \hfill \\ {\left. {u_{n} } \right|_{in} = \left. {u_{n} } \right|_{out} = V_{interface} } \hfill && {~~~~~~\left( {no - slip} \right)} \hfill \\ \begin{gathered} \left. {u_{\tau } } \right|_{in} = \left. {u_{\tau } } \right|_{out} \hfill \\ \mu_{in} \left. {\frac{{\partial u_{\tau } }}{\partial r}} \right|_{in} = \mu_{out} \left. {\frac{{\partial u_{\tau } }}{\partial r}} \right|_{out} \hfill \\ \end{gathered} \hfill && {\left. {\varvec{u}} \right|_{interface} = V_{interface} } \hfill \\ \end{array}$$

where **u**_**n**_ and ${\varvec{u}}_{{\varvec{\tau}}}$ are normal and tangential components of liquid velocity, ${\varvec{\mu}}$ viscosity, ${\varvec{V}}_{{{\varvec{interface}}}}$ velocity of the liquid–liquid or liquid–solid interface. Due to these boundary conditions in two-phase flows continuous phase induces two complex vortexes inside a moving droplet, which were recently measured by the micro-PIV technique^33^. If droplets are moving in a straight channel, the vortexes locate symmetrically to the center of the channel. Thus, reagent mixing is limited mainly by diffusion through the boundary between these vortexes.

The boundary conditions also play a key role in the structure of fluid flows during droplet formation. In a flow-focusing microfluidic device, it occurs in a jetting or dripping mode^34^. In the dripping mode, this process is divided into several stages: filling, necking, and pinch off^35^. During the filling stage, the velocity of the interface between two liquid phases is much smaller than the velocity of the continuous phase. Thus, the flow of the continuous phase induces vortexes in the dispersed phase during droplet formation. The symmetry of the channels determines the symmetry of these vortexes and the aperture inserted before the outlet channel increases the filling stage time to enhance the impact of these vortexes on the reagents distribution in newly formed droplets. Therefore, an asymmetric shape of the droplet generator induces an asymmetric vortex in the dispersed phase, which can improve mixing during the droplet formation.

**Figure 1:**
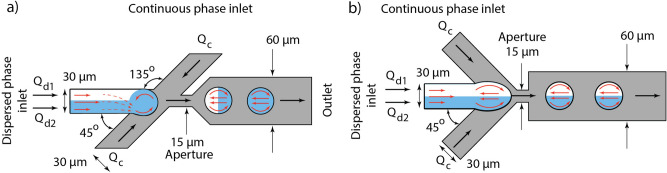
Schematics of fluid flows in the flow-focusing droplet generators. (**a**) The asymmetric design with side channels arranged at 45° and 135° to the central channel. In this design, the flow of the continuous phase induces a single vortex in the dispersed phase during the filling stage that enhances mixing. (**b**) The symmetric droplet generator with side channels arranged at the angle of 45° to the central channel. In this design, the flow of the continuous phase induces two vortexes in the dispersed phase and didn’t affect mixing.

To figure out how the symmetry of the channels influence on the fluids flows and reagents distribution in droplets, we considered an asymmetric flow-focusing microfluidic device with side channels arranged at 45° and 135° to the central channel (Fig. 1a), as well as a symmetric device with side channels arranged at 45° to the central channel (Fig. 1b). These devices have one inlet for the continuous phase (mineral oil) and two inlets for the dispersed phase (water and aqueous dye solution). The dye solution was introduced from the lower half of the dispersed phase channel (Q_d2_ at Fig. 1), while the other half was filled with water with zero concentration of the dye (Q_d1_ at Fig. 1). During and after droplet formation we investigated fluid flows and dye distribution inside the droplets using 2D numerical simulations and experimentally.

### Numerical simulations

Mixing efficiency during droplet formation directly depends on the velocity profiles of the continuous and dispersed phases in the microfluidic device. The key role in the mixing process plays the velocity and position of the interface between these phases. Several groups developed approaches to simulate droplet formation in symmetric flow-focusing devices^36,37^ and droplet breakup in the T-junction^38^. While 3D simulations provide the most accurate results of the process but require a lot of computing resources, 2D simulations can also provide relevant information about it and have a good qualitative agreement with the experimental data^36,39^. Moreover, it allows predicting droplets breakup in the T-junction and accurately simulate the early stages of the breakup, but can overestimate the neck thickness between two newly formed droplets^37^. Quantitative characterization of droplet generation regimes can be done if the geometry of the device allows using 2D axisymmetric simulations^37^.

To test how the symmetry of the flow-focusing region of the droplet generators influence fluid flows and reagents distribution we ran 2D numerical simulations of the droplet generation for both symmetrical and asymmetrical generators in Comsol Multiphysics using a similar approach to one described in^37^. Briefly, Navier–Stokes equations for incompressible fluids were solved to calculate the velocities. Fick's second law with the added convective term was used to model the distribution of a model dye. Displacement of the interface between two immiscible fluids was described by a phase field φ, which set the spatial distribution of the two phases and took values from -1 to 1. The isoline φ = 0 represented the liquid/liquid interface. To determine the phase-field, minimization of the system’s free energy was performed by solving the Cahn–Hilliard Eq. ^40^. The main advantage of the used phase-field method for simulation of two-phase flows is that it provides the opportunity to calculate contact line displacement with no-slip boundary condition for fluid velocity, reduces pressure jump at the corners, and prevents artificial vortex in the area of the channels crossing. Moreover, as an interface capturing method, it provides the opportunity to resolve droplet breakup. Thus, we were able to simulate the whole process of droplet formation. To obtain reasonably accurate results without a significant increase in computing requirements we chose mesh size 1 μm in a pinch region and 1.5 μm in the others (Fig. S1).

Although, as the interfacial profile evolves in a flow field, Cahn–Hilliard diffusion may shift the interface contour and effectively change the size of a drop. This leads to the escape of some reagent quantity from one phase to another one. To minimize such effect during the simulations the dye was injected in the opposite way that was shown in Fig. 1a. Such injection decreased the dye contact with the liquid–liquid interface. After the simulations, the concentration distribution was reversed to compare with experiments (see Material and methods, as well as Supplementary information, Fig. S2).

**Figure 2:**
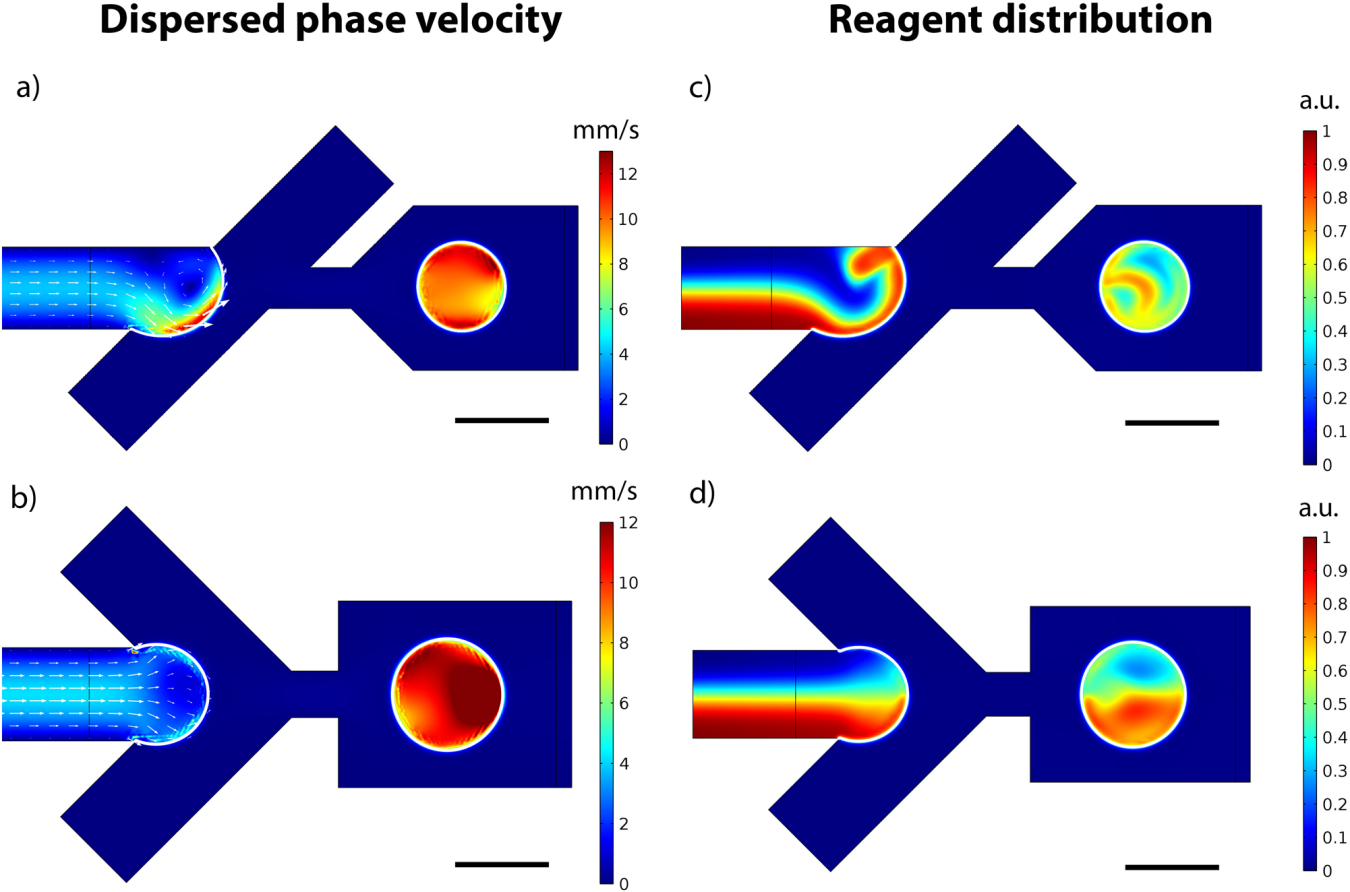
2D simulations of the droplet formation process. (**a**,**b**) Dispersed phase velocity profiles during the filling stage in the asymmetric and symmetric droplet generators. (**c**,**d**) Concentration distribution during droplet formation in the asymmetric and symmetric droplet generators. The continuous phase flow rate is 1 μl/min. The dispersed phase flow rate is 0.2 μl/min. The concentration is presented in arbitrary units according to the concentration of the reagent at the entrance boundary, which was set to 1 mol/m^3^. The scale bar is 30 μm.

The simulations of the droplet formation process show that during the filling stage velocity of the interface is much lower than the velocity of the continuous phase. This leads to the formation of one or two fluid recirculation vortices in the dispersed phase during the filling stage. These vortexes are induced by the continuous phase flow due to the boundary conditions on the liquid–liquid interface. In the asymmetric design, this single vortex is not symmetric to the output channel’s axis, therefore it can influence the reagents distribution inside droplets and enhance mixing (Fig. 2a,b, video V1). In the symmetric design, similar to what was reported previously^33^, there are two vortices located symmetrically that don’t enhance mixing (Fig. 2c,d, video V2). Moreover, fluid velocity in these vortices is up to twice higher in the asymmetric design than in the symmetric one. In contrast, the velocity of the interface during the necking and pinch off stages is comparable with the velocities of both phases, thus, these stages don’t affect mixing efficiency.

We found that the reagents distribution after droplet breakup strongly depended on the mobility tuning parameter in the Cahn–Hilliard equation. This parameter determines the time scale of the Cahn–Hilliard diffusion and can’t be derived from macroscopic parameters. Thus, we determined its value by comparing the simulation results with the experimental data and found that in our case the value 1 m∙s/kg showed good agreement over the wide range of flow rates (Fig. S3). Applying this value to our model we observed that the reagents distribution after droplet breakup in the asymmetric design is not uniform but the uniformity quickly increases due to the recirculating fluid flows inside moving droplets. Reagents distribution at other values of the mobility tuning parameter from 0.05 to 10 m s/kg are presented in Fig. S4.

To elucidate how obtained results depend on the absolute values of the flow rates we calculated the Reynolds number (Re) during the droplet generation. In the aperture Re ~ 1, while in the droplet formation area Re ~ 0.16. Thus, the flows were laminar but inertia forces could induce Dean flows. According to the previous studies the Dean flows should be taken into account if the Dean number (De) is about 10^41^. In the asymmetric geometry of the droplet generator, the Dean flows can appear in the droplet formation area due to the asymmetric vortex. In this vortex Re ~ 0.16 and ${\text{De}} \sim{\text{Re}}\sqrt {d/R}$ ~ 0.16, where *d* is the channel hydraulic diameter and *R* is the flow path radius of curvature. Thus, the Dean flows in our case may be neglected. However, if the flow rates are 100–1000 times higher the flows will be still laminar but the Dean flows may affect the velocity field distribution and further enhance mixing.

### Experimental studies

To experimentally investigate how the symmetry of the flow-focusing droplet generators influence fluid flows and reagents distribution we made microfluidic devices with symmetric and asymmetric designs from PDMS Sylgrad 184 by standard soft lithography technique^42,43^. Channels depths of these devices were 40 μm and 60 μm. Such depths corresponded to aspect ratios of droplet formation area 1:1 and 3:2 respectively and allowed us to test how accurate are the results of performed numerical simulations. Mineral oil was used as the continuous phase and water as the dispersed phase. Liquids were introduced into the devices by two syringe pumps (PHD 2000, Harvard Apparatus) from Hamilton syringes with volumes 500 μl and 100 μl. For micro-PIV and reagents distribution measurements 1 μm tracer particles or Coomassie Brilliant Blue G-250 were added into the dispersed phase.

**Figure 3:**
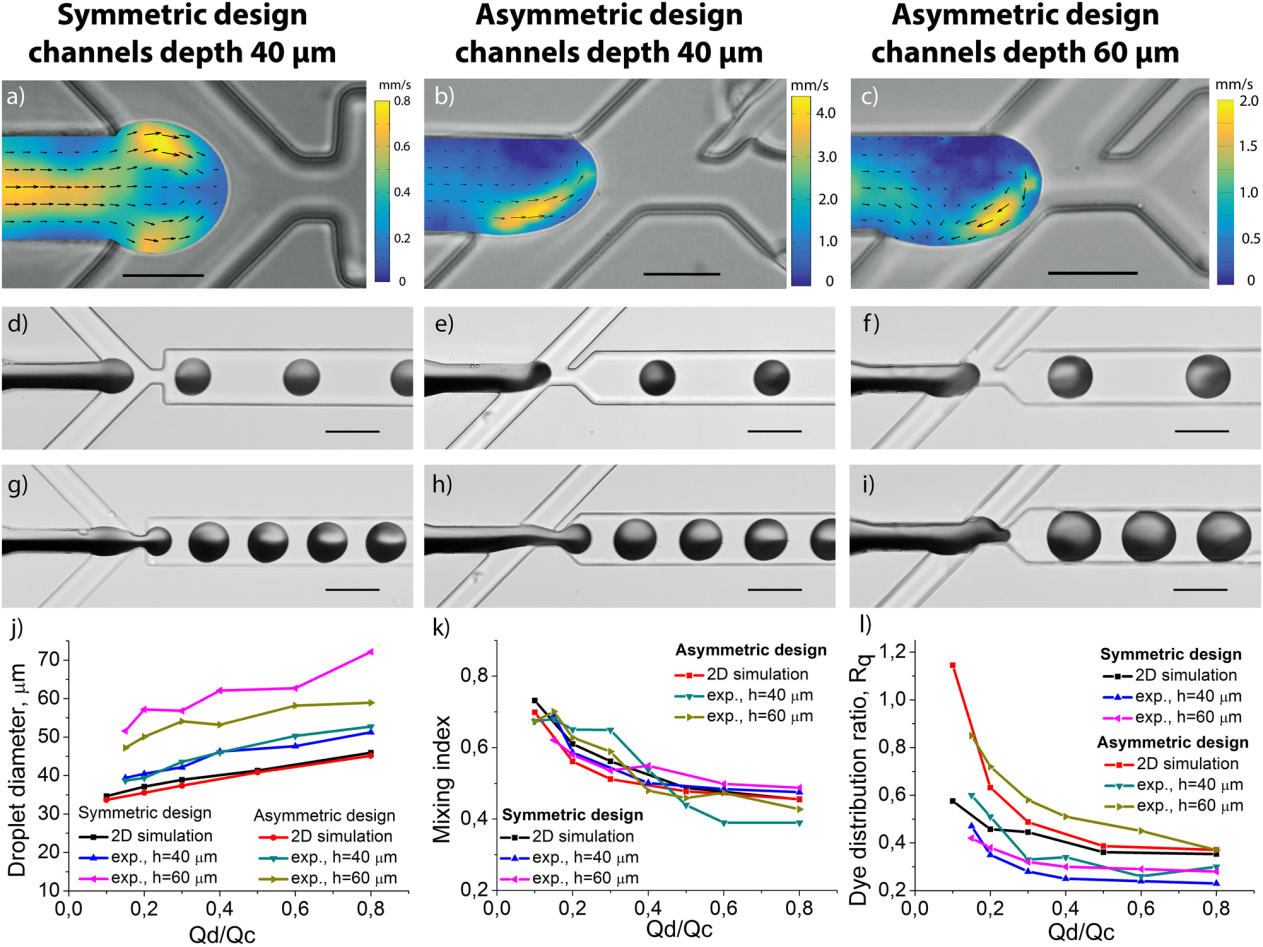
Experimental characterization of the droplet formation process. (**a**–**c**) PIV measurements of the dispersed phase velocity profile during the filling stage of the droplet formation process in symmetric and asymmetric generators. (**d**–**i**) Coomassie Brilliant Blue G-250 packaging into droplets in symmetric and asymmetric droplet generators. (**j**) Droplet diameters at different flow rates of the dispersed and continuous phases. (**k**) Mixing index in newly formed droplets in different microfluidic droplet generators. (**l**) Dye distribution ratio R_q_ at different flow rates. Dispersed phase and continuous phase flow rates Qd and Qc were (**a**–**f**) 0.2 μl/min and 1 μl/min, (**g**–**i**) 0.6 μl/min, and 1 μl/min, respectively. (**j**–**l**) Continuous phase flow rate was Qc = 1 μl/min. The scale bar is 30 μm in (**a**–**c**) and 60 μm in (**d**–**i**).

Two-dimensional micro-PIV measurements of the dispersed phase velocity profile showed that the direction and structure of the fluid flow in the droplet formation area during the filling stage depended on the symmetry and the channel depth of the device (Fig. 3, Fig. S5). To estimate the structure of the fluid flow and test if it was 2D or 3D we used the continuity equation for incompressible fluid div(V) = 0. According to this equation distribution of dimensionless value K = div(V_2D_)*h/|V_2D_|, where V_2D_ is the velocity obtained by 2D PIV, h is the channel’s depth, can indicate the three-dimensional structure of the flow, as it is proportional to the magnitude of the sinks and sources in the projected 2D flow field (Fig. S6). In the symmetric geometry, the fluid flow consisted of two symmetric vortexes with a sink in the center regardless of the channel aspect ratio. In contrast, in the asymmetric droplet generators, the structure and direction of the fluid flow strongly depended on the depth of the channels due to different positions of the interface between dispersed and continuous phases during the filling stage (Fig. 3a–c, Video V3–V6). The vortex attained a complex 3D flow structure with an increase in the aspect ratio from 1:1 (channel depth—40 μm) to 3:2 (channel depth h = 60 μm). This led to changing the fluid flow direction from counter-clockwise to clockwise regardless of the flow rates of the dispersed and continuous phases. Moreover, in both asymmetric geometries, these vortexes were asymmetric and their maximum velocity was several times higher than in the symmetric geometry, which corresponded to higher Taylor dispersion due to a higher Peclet number.

To determine how changes in the droplet generator’s geometry and fluid flows will influence the droplet parameters we measured their diameters and generation frequencies at various flow rates of the dispersed phase, while the flow rate of the continuous phase Qc was 1 μl/min (Fig. 3j, Fig. S7). In all cases, the generation regimes were stable with the coefficient of variation of the droplet diameter less than 4%. The measurements showed that they linearly depended on the ratio between the flow rates of the phases. If the aspect ratio of the droplet formation area is 1:1 (channel depth is 40 μm), the diameters and generation frequencies of droplets that were formed in the symmetric and asymmetric generators are close to each other and close to the simulation results. For the aspect ratio 3:2 (channel depth is 60 μm), droplet diameters and generation frequencies strongly depend on the device symmetry resulting in up to 30% larger diameter and higher frequencies in the symmetric geometry.

To investigate the mixing enhancement caused by the asymmetric vortexes in the droplet formation area we studied dye distribution in droplets during their traveling in the straight output channel. It was based on the measurements of light intensity inside the droplets that corresponded to the dye concentration and included calculations of the mixing index (MI)^44^ and the dye distribution ratio R_d_ between the its quantities in the top and bottom halves of the droplets:

2 $$MI = 1 - \left( {\frac{{\iint {(c - \overline{c})^{2} dA}}}{{A \cdot \overline{c}\left( {c_{max} - \overline{c}} \right)}}} \right)^{\frac{1}{2}} , R_{d} = \frac{{Q_{up} }}{{Q_{down} }},$$

where *A* is the droplet area, *c* is the concentration of the dye, $\overline{c}$ and $c_{max}$ are the average and the maximum concentrations of the dye within a droplet respectively, $Q_{up}$ and $Q_{down}$ are the dye quantities in the upper and lower halves of a droplet.

Dye distribution measurements (Fig. 3d–i) show that due to the asymmetric vortex the quantity of the dye in the upper half of the droplet increases. Immediately after the droplet formation, the mixing indexes are close to being similar for all considered droplet generators regardless of their design and channel depths (Fig. 3k). For small droplets with 35–45 μm diameter (30–50 pl volume), the mixing index is about 0.6–0.7 and decreases with the droplet size increase. In contrast, the distribution ratio *R*_*d*_ strongly depends on the symmetry of the droplet generators (Fig. 3l). In the asymmetric devices, it is significantly higher than in the symmetric ones. For small droplets (diameter 35–45 μm), *MI* ~ 1 and decreases while the volume of the droplet increases. For large droplets (diameter 45–60 μm) this ratio is similar for all of the devices. Such behavior indicates that direct mixing doesn’t occur during droplet formation due to the short duration of this stage. Indeed, the asymmetric vortexes change the reagents distribution in newly formed droplets and these changes are significant when droplets volume is close to the volume of the droplet formation area. Our further simulations show that these changes monotonically depend on the angle between the side channels and the central channel and are relatively strong because in asymmetric geometry the mixing index is almost independent of the diffusion coefficient in the range 10^–10^–10^–11^ m^2^/s, while in the symmetric geometry it rapidly decreases (see Fig. S8).

The direction and velocity field of the fluid flow in the droplet formation area of the asymmetric device with channel depth 40 μm (aspect ratio 1:1) is very similar to the simulation results. Surprisingly, with the depth increased to 60 μm (aspect ratio 3:2) the recirculating vortex shows the opposite direction. At the same time, experimentally determined values of mixing index and distribution ratio *R*_*d*_ in the devices with channels’ depth 60 μm are closer to the simulation results than for the devices with channels’ depth 40 μm. Such differences indicate that in our case 2D simulations are appropriate only for qualitative analysis.

**Figure 4:**
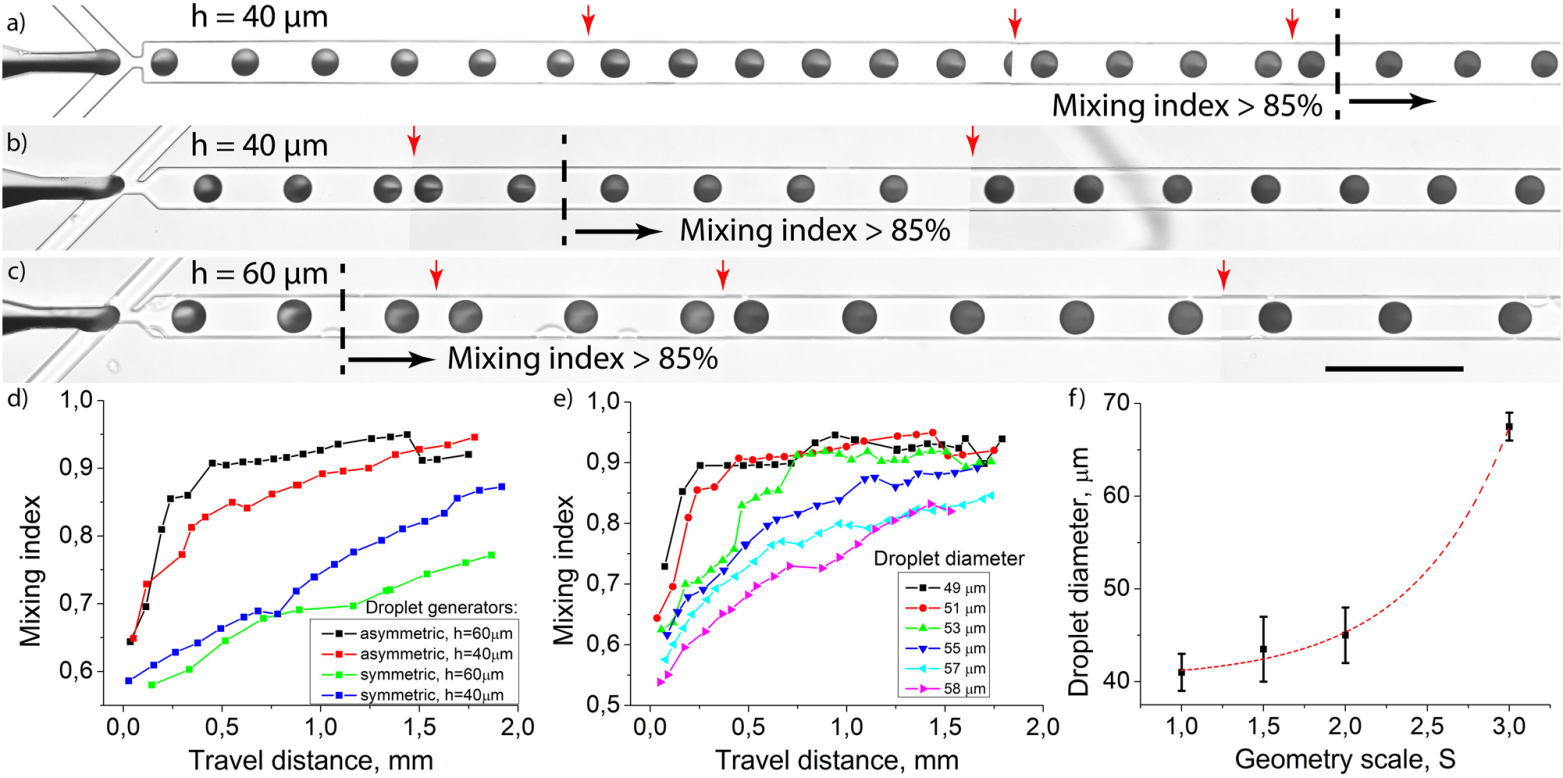
Evolution of the dye distribution during droplet migration in the straight output channel. Droplet generation and Coomassie Brilliant Blue G-250 packaging in the flow-focusing droplet generators with (**a**) symmetric geometry and the channel depth h = 40 μm, (**b**) asymmetric geometry and the channel depth h = 40 μm, (**c**) asymmetric geometry and the channel depth h = 60 μm. (**d**) Mixing index evolution during droplet migration in the straight output channel in different droplet generators (Qc = 1 μl/min, Qd = 0.2 μl/min). (**e**) Mixing index evolution inside droplets of different diameters formed in an asymmetric droplet generator with channels depth h = 60 μm. (**f**) Droplet diameter range with > 85% mixing efficiency after 0.6 mm travel in the output channel with different scaling of the droplet formation area S. Red dashed line is an eye guide. The scale bar (**a**–**c**) is 200 μm. Images (**a**–**c**) are mosaic images captured one after another in a single experiment, the red arrows indicate the stitching points.

After formation, the droplets started migrating along the straight channel to the outlet. During this migration, the reagents began mixing due to the recirculating fluid flows and diffusion inside the droplets. This led to a mixing index increase. Our experiments showed that the efficiency of this process depended on the initial reagents distribution directly after droplet formation (Fig. 4), in line with previous studies^30^. Thus, the symmetry of the flow focusing area, channel depth, and droplet volume directly affect it. In the case of droplets with 42 µm in diameter (volume 39 pl) formed in an asymmetric droplet generator with channels depth 40 µm, the generation frequency was 50 Hz and the mixing index of 0.85 was observed after 60 ms when droplets passed 0.55 mm in the strait output channel (Fig. 4a). If the depth of the channels was 60 μm, due to the 3D flows the mixing time was up to 0.85 level in 51 μm droplets (volume 69 pl) in 30 ms when droplets passed 0.24 mm in the output channel (Fig. 4b). In the case of the symmetric design with channels height 40 μm, we observed the same results only after 200 ms when droplets 40 μm in diameter passed 1.7 mm in the output channel (Fig. 4c). In the symmetric design with channels height 60 μm and after passing 2 mm in the output channel, the mixing index was only 0.8 for the smallest droplets with 47 μm in diameter.

The optimal volume of droplets for effective mixing can be defined as the volume of the droplet formation area. After increasing their size mixing efficiency decreases regardless of the channel depth (Fig. 4d,e, Fig. S9). If the depth is 60 μm (aspect ratio is 3:2) and droplet diameter increased from 50 to 55 μm (volume 62 pl to 83 pl) the improved mixing effect of the asymmetric geometry almost neglects (Fig. 4e). Thus, for effective mixing droplet diameter should vary less than 10% from the optimal value. In this case, volume variations can be 30%. We found similar relations for droplet generators with the channel depth of 40 μm (Fig. S10).

In comparison with the symmetric geometry (Fig. 4d, Fig. S10), for small droplets, the mixing efficiency is much higher in the asymmetric devices, although for large droplets it becomes similar. According to this, the asymmetric geometry provides effective reagent mixing in a 30% range of possible droplet volumes. To overcome this limitation, scaling of the droplet generation area may be used, while the whole size of the microfluidic device and fluidic connectors’ locations can be kept the same. We tested four droplet generators with different scaling of the droplet formation area with equal depth of the channels and found that diameters of droplets where effective mixing occured increased with the scaling factor *S* = *A*_2_/*A*_1_, where *A*_*i*_ is the square of the droplet formation area (Fig. 4f). We found that scaling the geometry 3 times caused the generation of droplets with up to 4 times larger volumes with effective reagent mixing. This increase is nonlinear due to the complex behavior of the droplet size depending on the geometry, aspect ratio, and the capillary numbers^34,45^. Thus, for each droplet diameter, the geometry of the droplet formation area should be determined individually to achieve the most efficient mixing.

## Conclusions

Here we developed, numerically simulated, and experimentally characterized a novel asymmetric design of flow-focusing droplet generator. This design enables the formation of a single asymmetric recirculation vortex in the dispersed phase during the droplet formation stage, which leads to an improvement in mixing speed. We found that the direction and velocity field of this vortex directly depends on the aspect ratio of the flow focusing area. For a 1:1 ratio (channels depth 40 μm), the vortex is counter-clockwise and mostly two-dimensional, which is very close to the simulation results. If the aspect ratio increases up to 3:2 (channels depth 60 μm) the interface between the continuous and dispersed phase changes its location, which leads to changing of the flow direction to clockwise, and the flow becomes predominantly three-dimensional.

We unraveled that the mixing index directly after droplet formation doesn't depend on the design of the droplet generator. Strikingly, due to the different initial distribution caused by the asymmetric vortex reagents mixing occurs up to six times faster in the asymmetric flow-focusing device than in the conventional symmetric one. The limitation of the proposed design is that the effective mixing occurs only in a narrow range of droplet volumes, which are close to the volume of the droplet formation area. To overcome it the droplet formation area can be scaled up to achieve the required droplet sizes.

Our studies showed that 2D numerical simulations can provide useful qualitative information about two-phase flows and droplet generation processes in quasi-two-dimensional devices. The relative simplicity of such simulations allows them to be easily applied to fairly complicated microfluidic geometries. The asymmetric design of the flow-focusing droplet generators, suggested in this work, provides significantly better mixing efficiency in comparison with the designs without additional mixing elements described before. We believe that the asymmetric design can find numerous applications and improve existing microfluidic devices for precise enzyme kinetic measurements, monitoring of different processes in real-time, and point-of-care devices, where reduction of the reagents mixing time and device simplicity is critical.

## Materials and methods

### Numerical simulations

Two-dimensional simulations of the droplet formation process in flow focusing microfluidic devices were performed in COMSOL Multiphysics (COMSOL Inc., Burlington, MA) using “Laminar Two-Phase Flow, Phase Field” and “Transport of Diluted Species” modules. In these simulations water with ρ_d_ = 1000 kg/m^3^ and dynamic viscosity μ_d_ = 0.001 Pa∙s was considered as the dispersed phase and oil with density ρ_c_ = 840 kg/m^3^ and dynamic viscosity μ_d_ = 0.03 Pa∙s was considered as the continuous phase. The flow rate of the continuous phase Q_c_ was varied from 0.1 μl/min to 10 μl/min at capillary numbers Ca = 0.01–1, and the flow rate of the dispersed phase Q_d_ was varied from 0.01 to 1 μl/min at capillary numbers Ca = 0.005–0.5. For these capillary numbers, droplet generation should occur in the dripping mode^34^. The surface tension σ at the oil–water boundary was set to 0.05 N/m without taking into account the surfactant, because it covered the interface and stabilizes droplets in several milliseconds after their formation^46^. The diffusion coefficient was set to 3.5 * 10^–10^ m^2^/s, which corresponded to the diffusion coefficient of small molecules and fluorescent dyes with similar molecular weights such as sugar, Cy5, Alexa 647, Alexa 633, and others^47–49^.

Navier–Stokes equations for incompressible fluid were solved to calculate the velocity. Fick's second law with the added convective term was used for modeling the distribution of reagent concentration. Displacement of the interface between two immiscible fluids was described by a phase field, which set the spatial distribution of the two phases and took values from − 1 to 1. To determine the phase-field minimization of the system’s free energy was performed by solving the Cahn–Hilliard Eq. ^40^. The mesh size was chosen 1 μm in a pinch region and 1.5 μm in the others after the convergence studies (154,949 degrees of freedom) (Fig. S1). For a detailed description of the simulation procedure please see the supplementary information.

For the Navier–Stokes equations at the entrance boundaries average velocities of the dispersed and continuous phases were defined according to the desired flow rates and the thickness of the channels, which was 40 μm in all the simulations. At the outlet boundary zero pressure was set. For the convection–diffusion equations at the half of the entrance boundary of the central channel (Fig. 1) reagent of unit concentration 1 mol/m^3^ was set. The other half was matched to the buffer with zero concentration of the reagent. At the outlet boundary the reagent flux was set to zero.

### Experimental

Microfluidic flow-focusing droplet generators were made by standard soft lithography technique^42,43^. At first, a single layer SU-8 photoresist mold was fabricated using contact optical lithography with a chromium mask. The PDMS prepolymer and the curing agent (Sylgrad 184, Dow Corning) were mixed in a ratio of 10:1 w/w, degassed, poured into the mold, and cured at 65 °C for 4 h in an oven. After the curing step, the PDMS replica with punctured inlet and outlet holes was bonded with cover glass slides by oxygen plasma treatment. Inlets for continuous and dispersed phases contained 5 μm and 20 μm filters, respectively, to prevent clogging. Channels depth was 40 μm and 60 μm, which corresponded to the aspect ratios of droplet formation area 1:1 and 3:2 respectively. The aspect ratios were calculated using the following equation: $Aspect Ratio = \frac{D}{\sqrt A }$ , where *D* is the channels depth and *A* is the square of the droplet formation area. All the inner surfaces of the channels were covered with a commercially available hydrophobic coating AntiRain Repellent (Turtle Wax, USA) to achieve the contact angle ~ 100°.

Light mineral oil (Sigma Aldrich, cat. No M8410) of density 840 kg/m^3^, viscosity 0.03 Pa*s with 3.5% w/w ABIL EM 180 surfactant (Evonik Nutrition & Care GmbH) was used as the continuous phase. MilliQ DI water was used as the dispersed phase. For fluid velocity measurements, 1 μm tracer particles (Polysciences Inc., cat. No 08226-15) were added into the dispersed phase. To study mixing of small molecules Coomassie Brilliant Blue G-250 (Sigma-Aldrich, cat. No 1154440025) was added into one of the dispersed phases.

The continuous and one or two dispersed phases were loaded into the Hamilton syringes with volumes 500 μl and 100 μl respectively, inserted into two syringe pumps (PHD 2000, Harvard apparatus, USA), and introduced into a microfluidic droplet generator at constant flow rates in the range 0.1–2 μl/min.

High-speed image acquisition (1000–6000 fps) was done by SC-1 camera (Edgertronic, USA) coupled with an inverted optical microscope Nikon Eclipse TE2000 with a 40 × NA 1.4 objective lens. PIV analysis was performed by the PIV lab software^50^. To obtain static images of droplets with Coomassie Brilliant Blue G-250 a 20 × NA 0.75 objective lens and a Nikon D3000 camera were used. The focus of the lens was adjusted at the center of the outlet channel of the device according to the maximum contrast of the droplets border. To calculate the mixing index inside droplets all color images were converted to black and white extracting data from the red channel. The red channel was chosen according to the absorption of the dye, which was in the range of 550–700 nm^51^. Light intensity inside droplets was used to calculate relative dye distribution and the mixing index by a custom Matlab script using Eq. (2). Briefly, all the droplets were manually selected from the images. Then for each droplet maximum and average concentrations of the dye were calculated analyzing the color of each pixel. The image of a single droplet was about 250 × 250 px^2^, the 20 px border of the droplets was ignored due to the high light scattering on the curved surface of the interface and interaction with the channel borders. To make the calculations more precise we inverted the images and normalized the intensity of the pixels inside droplets to the intensity of the pixels with zero and maximum dye concentration in the inlet channel before the droplet formation area. After this normalization, all the pixels had values from 0 to 1.

## Supplementary Information

Supplementary Information 1.

Supplementary Video 1.

Supplementary Video 2.

Supplementary Video 3.

Supplementary Video 4.

Supplementary Video 5.

Supplementary Video 6.
