# Supplementary material for: An asymmetric flow-focusing droplet generator promotes rapid mixing of reagents
Source: Sci Rep. 2021 Apr 22;11:8797. doi: 10.1038/s41598-021-88174-y (PMC8062447; doi:10.1038/s41598-021-88174-y)
Supplement: Supplementary file 1 — Supplementary Information 1. [file 41598_2021_88174_MOESM1_ESM.pdf]

An asymmetric flow-focusing droplet generator promotes rapid mixing of reagents.

K.I. Belousov[1], N.A. Filatov[1], I.V. Kukhtevich[2,3], V. Kantsler[4], A.A. Evstrapov[5], A.S. Bukatin[1,5]

Supplementary information

Numerical simulations detailed description

The simulations were performed by COMSOL Multiphysics software for solving differential equations based on the finite element method on a personal computer. Phase Field and Transport of Diluted Species interfaces were used for describing the evolution of the two-phase system and reagent concentration distribution. Phase Field interface used the Navier-Stokes equations for the simulation of fluids velocity fields:

$$\rho \frac{\partial \mathbf{u}}{\partial t} = \nabla \cdot \left[-p\mathbf{I} + \mu \left(\nabla \mathbf{u} + (\nabla \mathbf{u})^T \right) \right] + \mathbf{F}_{st} \quad (1)$$

$$\nabla \cdot \mathbf{u} = 0 \quad (2)$$

where

- ρ is the density (kg/m^3);
- **u** is the velocity vector (m/s);
- p is the pressure (Pa);
- **I** is the identity matrix;
- μ is the dynamic viscosity (Pa·s);
- $\mathbf{F}_{st}$ is the surface tension force (N/m^3).

The spatial distribution of the two phases was described by a phase field ϕ, which takes values from -1 to 1. To determine the phase-field evolution minimization of the system's free energy was performed by the Cahn-Hilliard equation, which is a 4th-order PDE. The Phase Field interface decomposed the Cahn-Hilliard equation into two second-order PDEs using help variable ψ:

$$\frac{\partial \phi}{\partial t} + \mathbf{u} \cdot \nabla \phi = \nabla \cdot \frac{\gamma \lambda}{\varepsilon^2} \nabla \psi \quad (3)$$

$$\psi = -\nabla \cdot \varepsilon^2 \nabla \phi + \left(\phi^2 - 1 \right) \phi \quad (4)$$

The quantity λ (N) is the mixing energy density and ε (m) is a capillary width that scales with the thickness of the interface. ε was set as a half of the maximum mesh size. These two parameters are related to the surface tension coefficient, σ (N/m), through the equation

$$\lambda = \frac{3\varepsilon\sigma}{\sqrt{8}} \quad (5)$$

and mobility γ (m^3·s/kg) is related to ε through

$$\gamma = \chi \varepsilon^2 \quad (6)$$

where χ (m·s/kg) is the mobility tuning parameter.

The surface tension force was added to the Navier-Stokes equations as a body force by multiplying the chemical potential of the system *G* by the gradient of the phase field variable:

$$\mathbf{F}_{st} = G\nabla\phi \tag{7}$$

$$G = \frac{\lambda\psi}{\varepsilon^2} \tag{8}$$

Transport of Diluted Species interface was added to the consideration by solving convection-diffusion equation:

$$\frac{\partial c}{\partial t} + \mathbf{u}\cdot\nabla c = \nabla\cdot\left(D\nabla c\right) \tag{9}$$

where *c* is the concentration of a reagent (mol/m^3), *D* is the diffusion coefficient (m^2/s), **u** is the velocity vector (m/s) calculated from (1) and (2).

To improve the quality of the obtained results quadratic shape functions were used to interpolate **u**, *c*, φ and ψ between the mesh nodes, and a linear shape function was used for pressure interpolation *p*. We used triangular mesh as the design of the droplet generator had acute and obtuse angles. The study of convergence with the decrease of the mesh size was conducted for asymmetric design with Q_d = 0.2 and Q_d = 0.7 µl/min. In both cases Q_c = 1 µl/min and D = 3.5·10^{-10} m^2/s (fig. S1). Mesh size equal 1 µm in a pinch region and 1.5 µm in others (154949 degrees of freedom) provided reasonably accurate results without significant increase in computing requirements. So we have chosen it for the simulations.

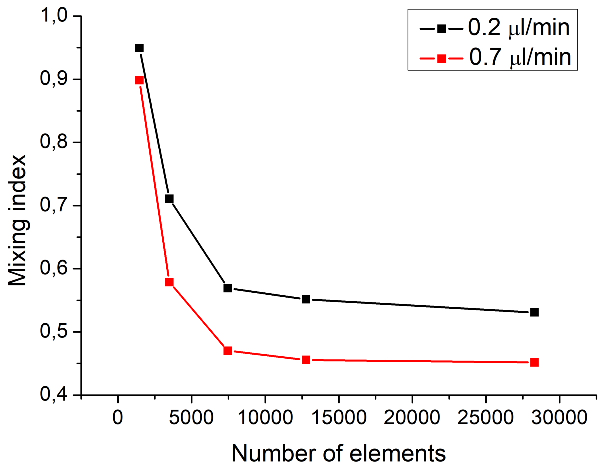

Figure S1. Mixing index obtained during simulation for convergence study. Q_d = 0.5, Q_c = 1 ul/min and D = 3.5·10^{-10} m^2/s

The advantage of the phase field method is that it provides the opportunity to calculate contact line displacement with no slip boundary condition for fluid velocity. It reduces pressure jumps at the corners and prevents artificial vortex in the area of channel crossing. Also the phase field method, as an interface capturing method, provides the opportunity to resolve droplet breakup, but Cahn–Hilliard diffusion may shift the interface contour and effectively change the size of a drop. This leads to escape of some reagent quantity from one phase to another. To minimize such effect the reagent was injected

along the longer side of the channel decreasing its contact with liquid-liquid interface (fig. S2a). Then concentration distribution was reversed to compare with the experiments (fig. S2b).

a) b)

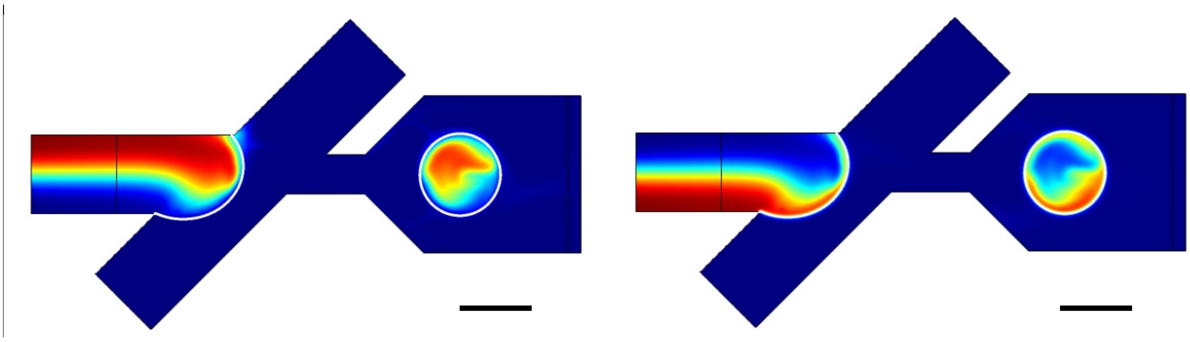

Figure S2. Reagent fraction distribution: a) during computation; b) after reversion in dispersed phase. Continuous phase flow rate is 1 µl/min, dispersed phase flow rate is 0.2 µl/min, mobility tuning parameter is 0.1 m·s/kg. The scale bar is 30 µm.

Another issue is the dependency of the results on the mobility tuning parameter. Mobility determines the time scale of the Cahn-Hilliard diffusion. It can't be derived from macroscopic parameters and should be chosen through the comparison with experimental results. The experiment with asymmetric design, where the continuous phase flow rate was 1 µl/min, the dispersed phase flow rate was 0.2 µl/min and channel depth was 60 µm was used as a benchmark. The mobility tuning parameter 1 m·s/kg was chosen as it showed good agreement both for the mixing index and for the dye distribution ratio R_q (fig. S3). Also this value provided the results consistent with our experiments on the wide range of flow rates. To indicate how the value of the mobility tuning parameter influences the dye distribution in newly formed droplets we made numerical simulations in newly formed droplets calculated at different values of the mobility tuning parameter are shown in figure S4.

a) b)

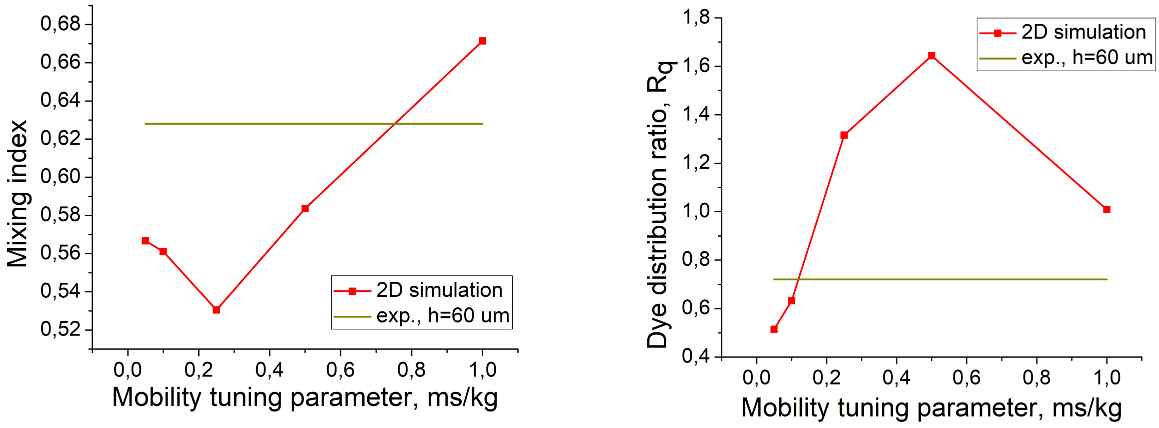

Figure S3. Simulation results at different mobility tuning parameters: a) mixing index; b) dye distribution ratio R_q. For these simulations the asymmetric design was used. The continuous phase flow rate was set to 1 µl/min, dispersed phase flow rate was 0.2 µl/min.

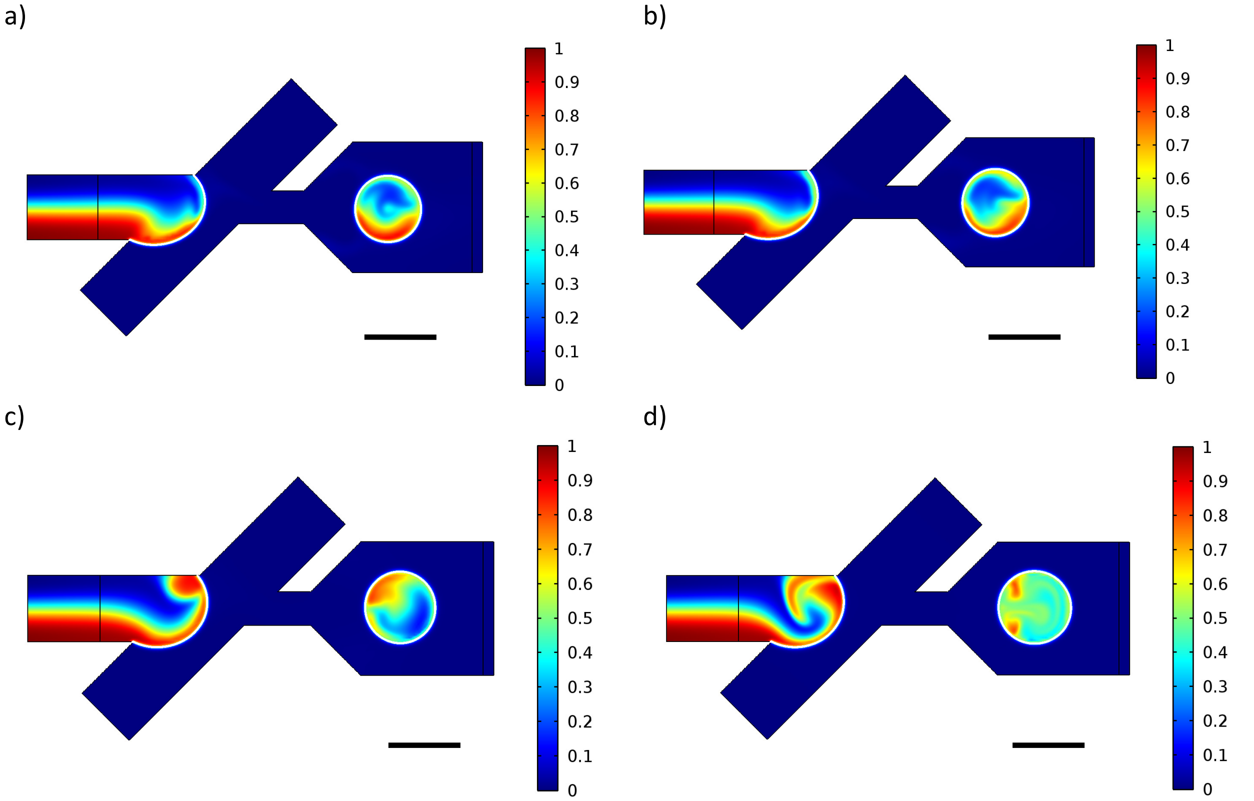

Figure S4. 2D simulations of the droplet formation process in the asymmetric flow focusing droplet generator at different values of the mobility tuning parameter: a) 0.05 m·s/kg, b) 0.1 m·s/kg, c) 0.5 m·s/kg, d) 10 m·s/kg. The scale bar is 30 µm.

Additional experimental and simulation results

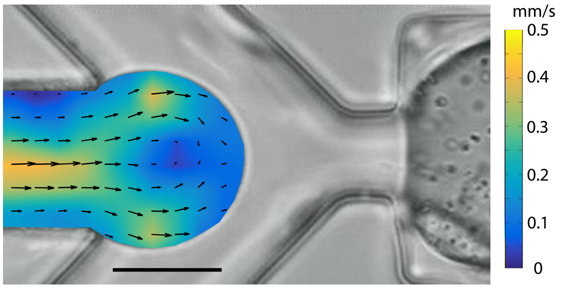

Figure S5. PIV measurements of dispersed phase velocity profile during the filling stage of droplet formation process in the symmetric microfluidic droplet generator with channel thickness 60 µm. Dispersed phase and continuous phase flow rates were 0.2 µl/min and 1 µl/min. The scalar bar is 30 µm.

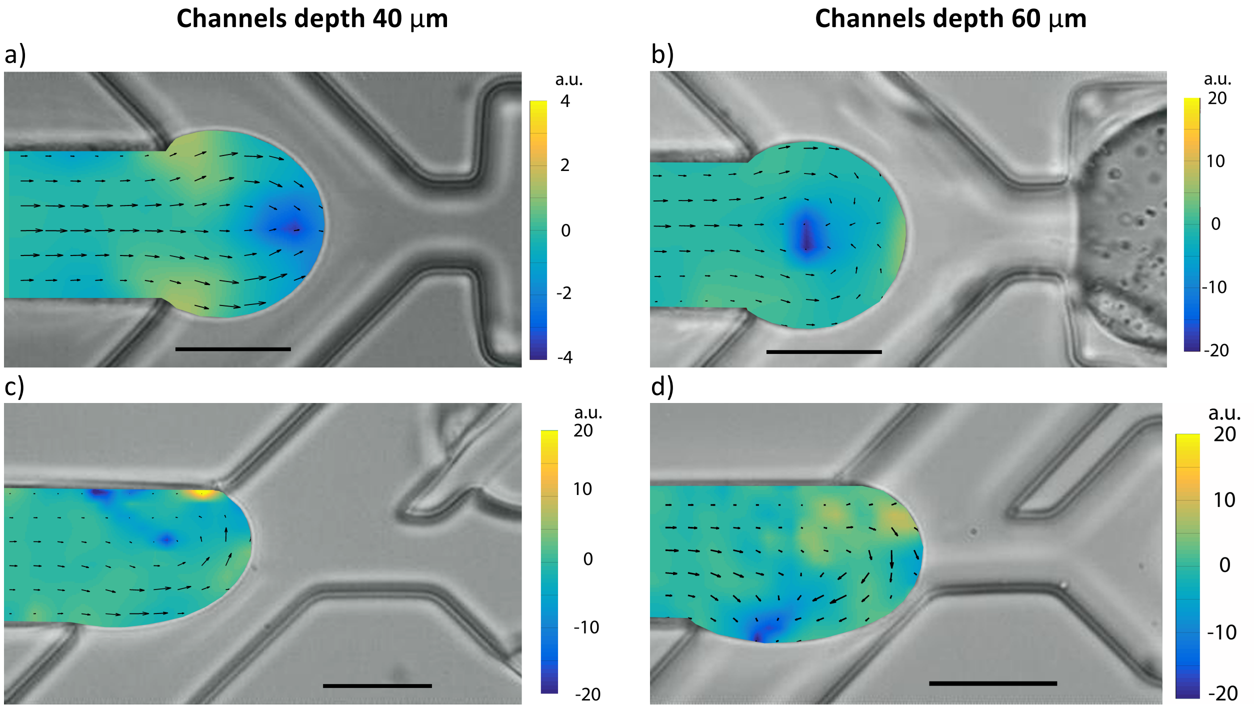

Figure S6. Distribution of the dimensionless value $K = div(V_{2D})*h/|V_{2D}|$ that characterize three dimensional flow during the filling stage of droplet formation, where V_{2D} – 2D velocity of the flow, measured by PIV; h – channels depth. a) Symmetric geometry, channels depth is 40 µm, b) symmetric geometry, channels depth is 60 µm, c) asymmetric geometry, channels depth is 40 µm, d) asymmetric geometry, channels depth is 60 µm. The scale bar is 30 µm.

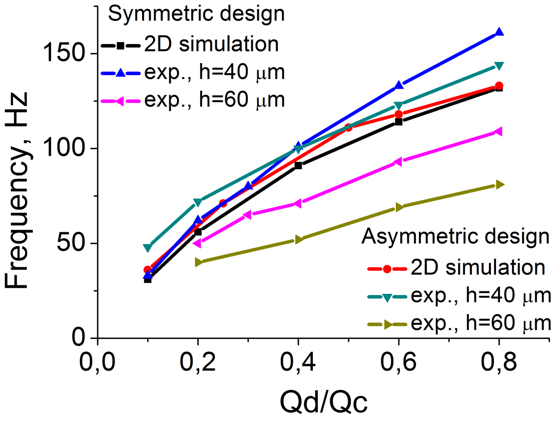

Figure S7. Droplet generation frequency at different flow rates of the dispersed phase Q_d, continuous phase flow rate was Qc = 1 µl/min.

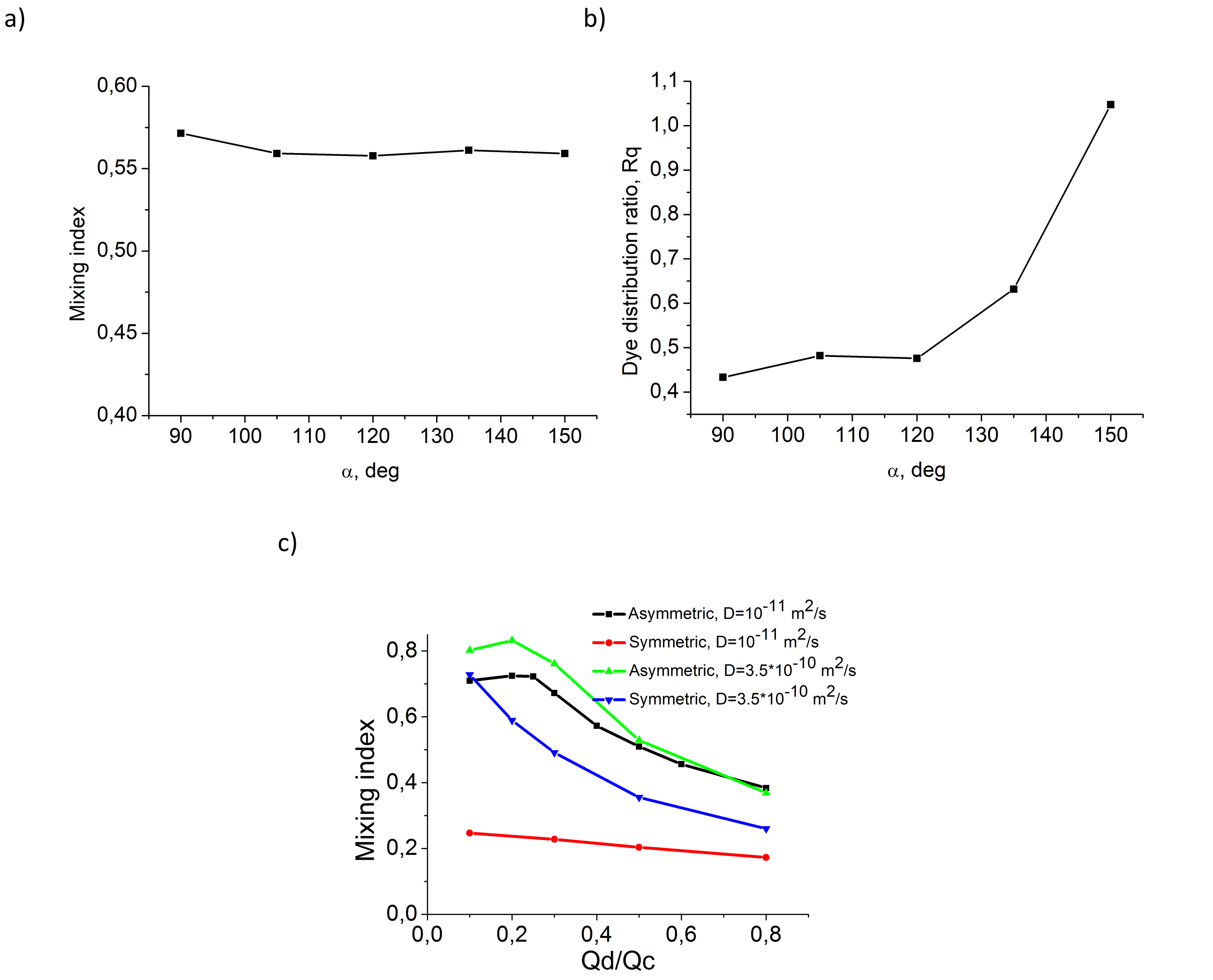

Figure S8. Dependence of the regent distribution inside a droplet after droplet formation on the largest angle α between the side channels and the central channel in the asymmetric device: a) mixing index, b) dye distribution ratio Rq; c) mixing index simulations direct after droplet formation in symmetric and asymmetric microfluidic droplet generators for reagents with different diffusion coefficients.

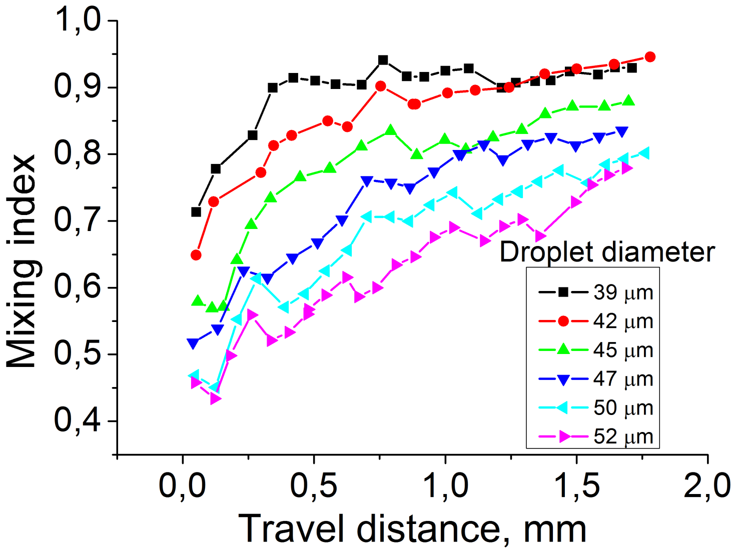

Figure S9. Mixing index evolution inside droplets of different diameters formed in an asymmetric droplet generator with channels depth h = 40 μm;

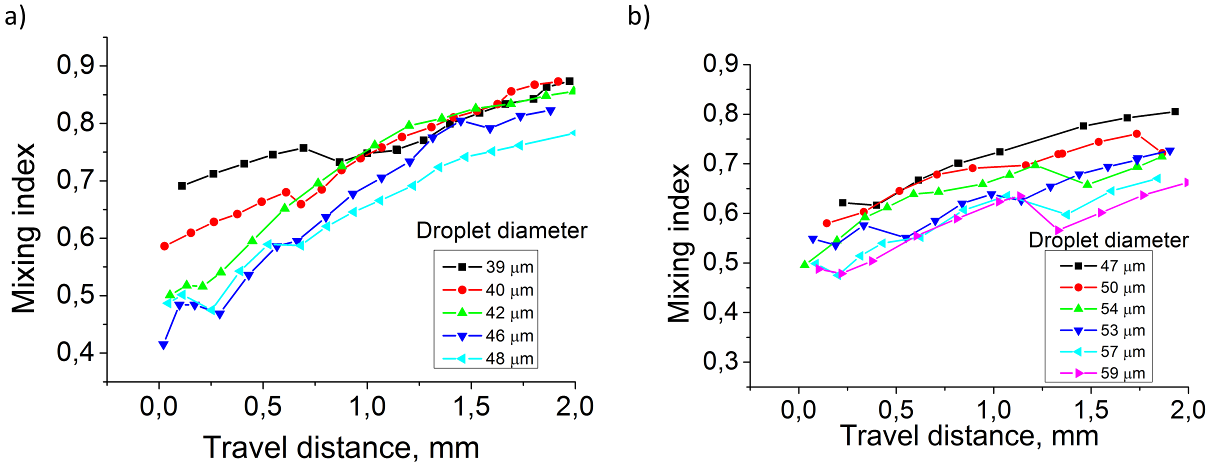

Figure S10. Mixing index evolution inside droplets of different diameters formed in a symmetric droplet generator with channels depth a) h = 40 μm; b) h = 60 μm.

Video captions

Video V1. 2D simulation of a droplet formation process: disperse phase velocity profile (top) and during concentration distribution (bottom) during droplet formation in an asymmetric droplet generator. Continuous phase flow rate is 1 μl/min, dispersed phase flow rate is 0.2 μl/min. The scalar bar is 30 μm.

Video V2. 2D simulation of a droplet formation process: disperse phase velocity profile (top) and concentration distribution (bottom) during droplet formation in a symmetric droplet generator. Continuous phase flow rate is 1 μl/min, dispersed phase flow rate is 0.2 μl/min. The scalar bar is 30 μm.

Video V3. Droplet formation process in an asymmetric droplet generator with channels depth h = 40 μm. Disperse phase contains 1 um tracer particles for PIV measurements. Continuous phase flow rate is 1 μl/min, dispersed phase flow rate is 0.2 μl/min. The scalar bar is 30 μm.

Video V4. Droplet formation process in an asymmetric droplet generator with channels depth h = 60 μm. Disperse phase contains 1 um tracer particles for PIV measurements. Continuous phase flow rate is 1 ul/min, dispersed phase flow rate is 0.2 μl/min. The scalar bar is 30 μm.

Video V5. Droplet formation process in an symmetric droplet generator with channels depth h = 40 μm. Disperse phase contains 1 μm tracer particles for PIV measurements. Continuous phase flow rate is 1 μl/min, dispersed phase flow rate is 0.2 μl/min. The scalar bar is 30 μm.

Video V6. Droplet formation process in an symmetric droplet generator with channels depth h = 60 μm. Disperse phase contains 1 um tracer particles for PIV measurements. Continuous phase flow rate is 1 μl/min, dispersed phase flow rate is 0.2 μl/min. The scalar bar is 30 μm.
